# RNAseq Analysis of Livers from Pigs Treated with Testosterone and Nandrolone Esters: Selection and Field Validation of Transcriptional Biomarkers

**DOI:** 10.3390/ani13223495

**Published:** 2023-11-13

**Authors:** Alessandro Benedetto, Kamil Šťastný, Nunzia Giaccio, Marianna Marturella, Elena Biasibetti, Maddalena Arigoni, Raffaele Calogero, Marilena Gili, Marzia Pezzolato, Kristína Tošnerová, Nikola Hodkovicová, Martin Faldyna, Roberto Puleio, Giancarlo Bozzo, Elena Bozzetta

**Affiliations:** 1 Istituto Zooprofilattico Sperimentale del Piemonte, Liguria e Valle d’Aosta, 10154 Turin, Italy; nunzia.giaccio@izsto.it (N.G.); marianna.marturella@izsto.it (M.M.); elena.biasibetti@izsto.it (E.B.); marilena.gili@izsto.it (M.G.); marzia.pezzolato@izsto.it (M.P.); elena.bozzetta@izsto.it (E.B.); 2 Department of Infectious Diseases and Preventive Medicine, Veterinary Research Institute, 621 00 Brno, Czech Republic; kamil.stastny@vri.cz (K.Š.); kristina.tosnerova@vri.cz (K.T.); nikola.hodkovicova@vri.cz (N.H.); martin.faldyna@vri.cz (M.F.); 3 Dipartimento di Biotecnologie e Scienze della Salute, Core-Lab di Bioinformatica e Genomica, Università degli Studi di Torino, 10124 Turin, Italy; maddalena.arigoni@unito.it (M.A.); raffaele.calogero@unito.it (R.C.); 4 Istituto Zooprofilattico Sperimentale della Sicilia, 90129 Palermo, Italy; roberto.puleio@izssicilia.it; 5 Dipartimento di Medicina Veterinaria, Università degli Studi di Bari Aldo Moro, 70121 Bari, Italy; giancarlo.bozzo@uniba.it

**Keywords:** transcriptomics, steroid esters, fattening pigs, PCA, qPCR

## Abstract

**Simple Summary:**

Testosterone and nandrolone can be illegally administered to meat-producing animals as synthetic esters. To tackle the abuse of growth promoters, alternative approaches able to investigate specific changes induced in proteins, transcripts, and metabolites are becoming recommendable. This work aimed to characterize transcriptome perturbations related to the illicit administration of steroid esters in fattening pigs. Animals were treated with testosterone esters (Sustanon^®^, Organon, Jersey City, NJ, USA) or nandrolone esters (Myodine^®^, Le Vet Beheer B.V., Oudewater, Utrecht, The Netherlands). At the end of the trial, liver samples were collected for gene expression studies. Comparisons between treated and control groups using RNAseq allowed the identification of 491 differentially expressed genes (DEGs). Further analysis of DEGs characterized a smaller cluster of 16 genes. A field survey performed on liver samples collected from pigs belonging to different breeds and weight categories allowed the validation of the selected biomarkers using qPCR, confirming their specificity by comparison with testosterone residue profiles on respective serum samples.

**Abstract:**

The use of anabolic–androgenic steroids (AASs) as growth promoters in farm animals is banned in the European Union, representing both an illicit practice and a risk for consumer health. However, these compounds are still illegally administered, often in the form of synthetic esters. This work aimed to characterize significant coding RNA perturbations related to the illicit administration of testosterone and nandrolone esters in fattening pigs. A total of 27 clinically healthy 90-day-old pigs were randomly assigned to test and control groups. Nine animals were treated with testosterone esters (Sustanon^®^) and other nine with nandrolone esters (Myodine^®^). At the end of the trial, liver samples were collected and analyzed using RNAseq, allowing the identification of 491 differentially expressed genes (DEGs). The transcriptional signature was further characterized by a smaller sub-cluster of 143 DEGs, from which a selection of 16 genes was made. The qPCR analysis confirmed that the identified cluster could still give good discrimination between untreated gilt and barrows compared to the relative testosterone-treated counterparts. A conclusive field survey on 67 liver samples collected from pigs of different breeds and weight categories confirmed, in agreement with testosterone residue profiles, the specificity of selected transcriptional biomarkers, showing their potential applications for screening purposes when AAS treatment is suspected, allowing to focus further investigations of competent authorities and confirmatory analysis where needed.

## 1. Introduction

The use of anabolic compounds as growth promoters in farm animals has been banned in the European Union since 1988, according to the Council Directive 96/22/EC. There have also been several indications regarding their adverse effects on animal and consumer health [1,2], and their illicit administration has been revealed by official control authorities in different farm species, as reported over the years by the European Food Safety Authority (EFSA) [3]. Despite the persistence of relatively low rates of non-compliant samples being reported for several classes of growth promoters (i.e., sex steroids, β-agonists, tyrostatics, etc.), in the more recent EFSA reports [4,5], parallel investigation activities carried out to contrast black market imports of banned drugs within the EU often reported recurrent seizures of prohibited steroids intended both for humans and animals [6,7]. Indeed, the refined use of low-dose cocktail preparations of some “old” and other more recent doping agents like designer drugs are often suspected to reduce the efficacy of official controls in place [8,9].

Moreover, not only synthetic steroidal compounds but also natural steroids can be illegally administered to fattening animals to speed up livestock productivity and reduce related meat production costs. In addition, endogenous occurrences of some anabolic steroids have been documented in different food commodities [10,11]. It is therefore difficult to distinguish between natural endogenous steroids, for which detectable physiological levels could be expected in analyzed samples, and residues of exogenous steroids resulting from illicit administration [12,13]. All these aspects often require additional investigations by inspection services for a reliable interpretation of lab results and, in many cases, it is not possible to discriminate between treated and untreated animals based on just the presence/concentration of these substances [14].

Consequently, the application of alternative approaches, based on biological methods, that are able to investigate perturbations induced by growth promoters at the level of proteins, transcripts, and metabolites in different tissue specimens and/or bodily fluids is becoming more and more widespread [15]. 

Considering large animals bred for meat production, the literature regarding steroid administration in pig farming is limited when compared with other farm species like cattle [16]. Currently available screening and confirmatory methods in the frames of the National Residue Control Plans (NRCPs) of EU member states are mainly based on targeted methods (ELISA, LC-MS/MS) for specific residue detection in selected tissues and/or biological fluids without considering the potential implementation of more recent omics technologies and untargeted High-Resolution Mass Spectrometry (HRMS). Some interesting applications of untargeted metabolomics for detecting the illicit use of growth promoters in pig farming have been recently reported [17,18,19,20], but other omics applications such as transcriptomics and proteomics are still missing in this species. Therefore, the aim of this work was to develop alternative screening approaches based on RNAseq characterization of fattening pig transcriptome perturbations related to the administration of known anabolic steroids like testosterone and nandrolone. The liver was chosen for being one of the main biotransformation sites of steroid compounds, already included in the official sampling programs at the abattoir of the Italian NRCP for other classes of forbidden growth promotes (e.g., β-agonists).

## 2. Materials and Methods

### 2.1. Experimental Animals

A total of 27 grower pigs (barrows, boars, and gilts), i.e., hybrids of Large White × Landrace (sow) × Duroc (boar), were bought from a local pig trader (Bioprodukt Knapovec, a.s., Ústí nad Orlicí, Czech Republic). All animals selected for the trial were clinically healthy and were acclimatized to the stable conditions until weaning; they were then randomly assigned to test (18 animals) and control (9 animals) groups. To assess potential confounding variance on studied hormonal treatments, sex ratios were balanced between different treated and untreated-control groups by considering equal numbers of castrated males (barrows), uncastrated males (boars), and female (gilts) specimens. Pigs were housed in separated pens of 2.8 × 2.0 m for each tested group and fed twice a day with a commercial diet according to their weight categories (DeHeus, a.s., Bučovice, Czech Republic). Animal monitoring was conducted at least two times a day for evaluation of welfare and health conditions according to national legislation (Act. no. 246/1992 Coll.). Animals from the control group were held in a separate room to achieve the maximum non-stressful conditions. Nine of the 18 animals from the test group were treated with intramuscular (i.m.) injections of 4 mg/kg b.w. (body weight) of the hormonal preparation 17β-testosterone (Sustanon 250 mg/mL; Organon, Czech Republic Reg. no. 56/357/91-C). The composition of Sustanon 250 mg/mL included a total amount of 176 mg/mL testosterone in the following constitution: Testosterone propionas 30 mg; Testosterone phenylpropionas 60 mg; Testosterone isocaproas 60 mg; Testosterone decanoas 100 mg; and benzyl alcohol and arachidis oleum in a non-specified amount. The other nine animals of the test group underwent i.m. injections of 5 mg/kg b.w. of the hormonal preparation 19nor-17β-testosterone ester (Myodine 25 mg/mL; Le Vet Beheer B.V., Czech Republic Reg. no. 96/030/17-C). The composition of Myodine 25 mg/mL included a total amount of nandrolone of 15 mg/mL as Nandrolone lauras 25 mg, benzyl alcohol 104 mg, and arachidis oleum in a non-specified amount. The reported concentrations of anabolic–androgenic steroids (AASs) were selected based on the results of the preclinical trial (see Section 2.1.1).

#### 2.1.1. Preclinical Trial

An acclimatization phase of 14 days was included prior to all procedures. After that, the initial weighing and group division was made via random selection of individuals based only on sex. The preclinical phase lasted a total of 43 days. The first single administration of testosterone and nandrolone to the experimental groups started on day 1 (D1) of the preclinical phase. To conduct a preliminary check of the absence of adverse and/or unexpected effects of the studied drugs, the testosterone group was treated with a single i.m. injection of 4 mg/kg b.w. of the hormonal preparation Sustanon (250 mg/mL) and the nandrolone group was treated with a single i.m. injection of 2 mg/kg b.w. of the hormonal preparation Myodine (25 mg/mL). Blood samples were collected from days 1, 2, 4, 7, 14, and 28 post-treatment to day 43 to determine the optimal dose of AASs from the pharmacokinetic curve in the following clinical study. 

#### 2.1.2. Clinical Trial

Based on results collected from the preclinical trial (data not shown), the applications of testosterone (administered dose fixed at 4 mg/kg b.w of Sustanon) and nandrolone (increased dose of Myodine up to 5 mg/kg b.w) to the experimental groups were then started on day 94 of the experimental phase. In total, five intramuscular (i.m.) applications of tested substances were made into the neck muscle, while with each application, the left and right side of the body were alternated. The injections were made on the following days: D94, D98, D102, D106, and D110. At the end of the experiment (day 114), animals were relocated to the facility of Steinhauser s.r.o., (Tisnov, Czech Republic) and authorized for commercial slaughter procedures according to current legislation (Council Regulation (EC) No. 1099/2009 on the protection of animals at the time of killing). Blood and tissue samples were then collected for further analyses. Specifically, 20 mg portions of liver samples were taken from the Spigelian lobe and stored in RNAlater solution (Thermo Fisher Scientific, Inc., Waltham, MA, USA), together with blood specimens for serum collection and subsequent steroid residue analysis. The control weighing of individuals was made at D92 and during the clinical phase at D105 and D113.

### 2.2. Field Survey

After the end of the trial on experimental animals, a field survey was carried out on pig slaughterhouses located in Italy. All selected facilities were authorized for pig slaughter procedures according to Regulation 1099/2009.

In order to assess potential sources of variability on investigated transcriptional biomarkers, blood/serum samples were collected together with liver specimens from selected animal batches considering all main industrial breeds (Landrace, Duroc, Large White, and commercial crossbred) and, when possible, some traditional Italian breeds (e.g., Black Pig of Sicily, Black Pig of Piedmont). Furthermore, three different finishing categories were considered during samplings:

- grower pigs with weight ranges lower than experimental animals (50–60 kg)
- medium-size pigs intended for fresh pork meat production (from 90 kg up to 110 kg)
- heavy-weight pigs intended for cured meat and production of sausages (from 150 kg up to 180 kg b.w.).

### 2.3. Molecular Analysis

#### 2.3.1. Sample Preparation

Total RNA was extracted from collected liver samples using RNeasy Plus Universal Mini Kit (Qiagen). RNA quality and quantity were checked using Qubit RNA BR kit (Thermo Fisher Scientific, Inc., Waltham, MA, USA) and analysis on Bioanalyzer 2100 (Agilent Technologies, Inc., Santa Clara, CA, USA). Samples with RNA Integrity Number (RIN) <7 were excluded from subsequent analyses.

#### 2.3.2. RNA Sequencing Analysis

To detect coding RNAs, the TruSeq stranded mRNA library preparation kit (Illumina Inc., San Diego, CA, USA) was used, following the manufacturer’s instructions. Briefly, 1 µg of total RNA was purified using poly-T oligo attached magnetic beads and then fragmented into small pieces using divalent cations at an elevated temperature (8 min at 94 °C). First-strand and second-strand cDNA were synthetized, a single A nucleotide was added to the 3’ ends of the blunt fragments, and multiple indexing adapters were ligated to the end of the double-stranded cDNA. PCR of 15 cycles was performed to selectively enrich the DNA fragments that had adapter molecules on both ends. Each library was analyzed with the DNA 1000 chip using Agilent 2100 Bioanalyzer (Agilent) and quantified using the Qubit DNA HS kit (Thermo Fisher Scientific, Inc.). A pool of all libraries (pooled at equi-molar concentration) was generated, quantified with Qubit DNA HS kit (Thermo Fisher Scientific, Inc., Waltham, MA, USA), and run at a concentration of 1.7 pM on the NextSeq500 sequencer (Illumina Inc., San Diego, CA, USA) in 75 nts paired end sequencing mode following the manufacturer’s instruction. RNAseq data are available in the Gene Expression Omnibus (GEO) data repository under the accession code GSE233177.

Fastq files were analyzed using the docker4seq package [21]. In brief, the quality of fastq files was evaluated with fastqc. Fastq files were trimmed with Skewer and mapped with STAR 2.5 against the *Sus scrofa* genome retrieved from the EMBL repository. Differential expression analysis was performed with DEseq2 [22]. Heatmaps were generated with Morpheus software (data were Z-score normalized and hierarchical clustering was performed using Euclidean distance and average linkage) (https://software.broadinstitute.org/morpheus/, access date: 6 November 2023).

Identified differentially expressed genes (DEGs) were filtered on the basis of thresholds applied on recorded fold changes (|log2FC| ≥ 1), with statistical significance (*p* < 0.05).

An enrichment analysis of DEGs was then performed to identify the most significantly enriched Gene Ontology (GO) biological process terms (BP), molecular function terms (MF) and cellular component terms (CC) using the gProfiler webserver [23], with the following settings: ordered query ranked by log2 fold change expression values, considering only annotated genes, with gSCS threshold < 0.05.

Further Gene Set Enrichment Analysis (GSEA) was performed using GSEA software (ver. 4.3.2, Broad Institute, Cambridge, MA, USA) to more accurately rank and quantify the association between the whole gene sets and the phenotypes of interest (Sustanon and Myodine treatments in our case) [24]. For this analysis, a preliminary conversion of pig gene IDs to relative human orthologs was made using the gProfiler Gene ID conversion tool, needed for accession to GSEA Molecular Signatures Databases (MSigDB), only available for human and mouse species.

#### 2.3.3. Biomarkers Validation Study

Confirmatory analysis of expression levels of filtered DEGs from RNAseq experiments was then performed using Real-Time PCR (qPCR), according to the ΔΔCq relative quantification method applied for proper transcriptional biomarker validation [25].

A preliminary evaluation of 14 reference genes was conducted using GeNorm analysis tools [26] embedded in CFX Maestro software v.2.3 (Bio-rad Laboratories Inc., Hercules, CA, USA) to select a panel of stable genes needed for suitable normalization of DEG expression levels in tested liver samples. The reference genes selected for stability tests were as follows: Elongation factor 1 alpha (EF1A), beta-2-microglobulin (B2M), hypoxanthine phosphoribosyltransferase 1 (HPRT1), hydroxymethylbilane synthase (HMBS), tyrosine 3-monooxygenase/tryptophan 5-monooxygenase activation protein zeta (YWHAZ), actin beta (BACT), ubiquitin B (UBB), aldolase fructose-bisphosphate A (ALDOA), TATA-box binding protein (TBP), Ribosomal protein L32 (RLP32), glyceraldehyde-3-phosphate dehydrogenase (GAPDH), DNA topoisomerase II beta (TOP2B), phosphoglycerate kinase 1 (PGK1), and peptidylprolyl isomerase A (PPIA).

All primers for selected DEGs and reference genes were designed using Primer Express Software 3.0 (ThermoFisher) and checked for specificity and secondary structures by both in silico mFold analysis [27] and in vitro melting profiling on different ten-fold serial dilutions of pooled liver RNA samples (from 1 µG to 1 nG of total RNA) to test linearity and efficiency of each assay, according to MIQE guidelines [28]. Briefly, 1 µG of total RNA extract from each liver sample was retrotranscribed into cDNA using iScript gDNA Clear cDNA Synthesis Kit (Biorad, Hercules, CA, USA) and then amplified with each selected primer pair using Real-Time PCR runs on a CFX96 touch thermal cycler (Biorad). The 1X of iTaq Universal SYBR Green Supermix (Biorad), 200 ng of each primer pair, and 1:5 dilutions of cDNA were mixed in 20 µL reactions. A dedicated thermal protocol, including melting curve stage, was then applied to each reaction: initial denaturation at 95 °C for 30 s, 40 cycles at 95 °C for 3 s plus annealing/extension step at 60 °C for 20 s, followed by melt curve stage from 65 °C to 95 °C with temperature increment of 0.5 °C every 5 s. Three extraction replicates for each sample were analyzed with two PCR technical replicates for all selected targets.

### 2.4. Experimental Samples Residues Analysis

Serum samples from experimental animals were collected during slaughter procedures and stored at −80 °C until analysis. Analysis of experimental specimens was performed to check at residue levels the successful application of AAS treatments under study.

#### 2.4.1. Chemicals and Reagents

Methanol, water, and formic acid were of analytical- or HPLC-grade quality and were supplied by Merck KGaA (Darmstadt, Germany). SPE columns OASIS HLB (60 mg, 3 mL) were purchased from Waters (Petit-Couronne, France). 19-nor-17β-testosterone (nandrolone), 17β-testosterone, and 17β-testosterone-d3 were supplied by Merck KGaA (Darmstadt, Germany). The stock-standard solutions of each analyte and internal standard (ISTD) were prepared in methanol at a concentration of 1 µg/mL and stored at −20 °C in the dark; solutions were stable for 2 years. Suitable working standard solutions in methanol were obtained via appropriate dilution of the corresponding stock solutions and stored at −20 °C.

#### 2.4.2. Blood Sample Preparation

Blood serum (1 mL) was spiked with 50 μL ISTD and diluted with 5 mL water/methanol (1:1). Samples were loaded onto Waters OASIS HLB SPE (60 mg, 3 mL), previously conditioned with 1 mL water and 1 mL methanol; the analytes were eluted with 2 × 1 mL methanol. After evaporation to dryness using a nitrogen stream at 50 °C, residues were dissolved in a 0.1 mL methanol/water (1:1) mixture.

#### 2.4.3. LC-MS/MS Analysis 

LC analysis was carried out through an HPLC system, Vanquish (Thermo Fisher Scientific, Inc.). Chromatographic separation was performed on a Phenomenex Kinetex (2.1 × 100 mm; 1.7 μm) column, kept in a column oven at 40 °C, using gradient elution with solution A (0.1% formic acid in water) and B (0.1% formic acid in water in methanol). 17β-testosterone-d3 was used as an internal standard. 

A Q Exactive mass spectrometer was used (Thermo Fisher Scientific) that was equipped with a heated electrospray ionization probe measured in a positive mode (H-ESI+). For the quantification analysis, the mass spectrometer worked in the parallel reaction monitoring mode PRM with high resolution RP = 17,500 (FWHM) at 200 *m*/*z*. The mass spectrometer was externally calibrated for mass accuracy using an ionic calibration solution (Thermo Fisher Scientific). The injection volume was 5 µL and the flow rate was 0.3 mL/min, with an overall run time of 10 min. The gradient profile was as follows: 0–1 min 50% (B); 1–5 min linear increase up to 95% (B); 5–7 min, 95% (B); 7–8 min, ramp linearly to 50% (B); and 8–10 min, 50% (B).

The method was validated both for screening and quantification purposes according to Commission Regulation (EU) 2021/808 in the following concentration range: 0.25–80 µg/L for 17β-testosterone, nandrolone, and their corresponding esters.

### 2.5. Analysis of Field Sample Residues

Serum samples from the field survey performed in abattoirs located in Italy were collected during slaughter procedures and stored at −80 °C until analysis. For analytical investigations, a fit-for-purpose validated method already implemented in bovine serum specimens [22] was applied to collected pig serum samples with minor modifications (see Section 2.5.1, Section 2.5.2 and Section 2.5.3).

#### 2.5.1. Chemicals and Reagents 

Analytical-grade quality acetonitrile, methanol, and ammonium fluoride were supplied by Sigma-Aldrich (St. Louis, MO, USA); molecularly imprinting polymer (MIP) SPE cartridges were purchased from Affinsep (Petit-Couronne, France). 17β-estradiol, 17β-estradiol-d4, 17β-testosterone, 17β-testosterone-d3, progesterone, and progesterone-d9 (Sigma-Aldrich, St. Louis, MO, USA) were used for standard solutions and ISTD.

#### 2.5.2. Blood Sample Preparation

Briefly, blood serum (2 mL) was spiked with ISTD and diluted in water. Samples were loaded on MIP SPE cartridges conditioned with acetonitrile and water; after washing and drying steps of the cartridge, analytes were eluted in methanol, then dried in a nitrogen stream and finally dissolved in 0.1 mL of methanol/water mixture.

#### 2.5.3. LC-MS/MS Analysis 

LC analysis was performed on Exion HPLC system (Sciex, Framingham, MA, USA) connected to a QTRAP 6500+ mass spectrometer (Sciex); two analytes of interest were detected in ESI-positive (17β-testosterone and progesterone) and the other in ESI-negative (17β-estradiol) Multiple Reaction Monitoring (MRM) mode. Chromatographic separation was developed on a Waters XSelect HSS T3 XP (3 × 100 mm; 2.5 μm) column, setting up a gradient elution with solution A (NH4F 0.2 mM in water) and solution B (NH4F 0.2 mM in methanol). Injection volume was 25 µL; the overall run time was 20 min. Detailed MRM conditions are shown in Table 1.

The developed method was limited only to the molecules included in the Italian National Residue Control Plan (NRCP) and, accordingly, validated for both screening and quantification purposes, with the following concentration ranges: 0.020–0.080 µg/L for 17β-estradiol, 0.25–10 µg/L for 17β-testosterone, and 0.5–20 µg/L for progesterone. 

### 2.6. Data Analysis

Statistical analyses were performed using Prism 7.0 software (GraphPad Software, Inc, Boston, MA, USA) and GenEX software 6.1 (MultiID Analyses AB, Frölunda, Göteborg, Sweden). Data were tested for normal distribution using Kolomogorov–Smirnov’s test. Comparisons and related statistical differences between controls and treated groups were assessed using the unpaired Student’s *t*-test (two-tail) for normally distributed data. Two-tail Mann–Whitney U-test was used for data that were not normally distributed. Comparisons were considered significantly different with *p* < 0.05.

A multivariate statistical approach was then applied to all collected data, based on the application of Principal Component Analysis (PCA) embedded in GenEX software, consisting of a first pattern recognition analysis followed by a classification analysis. PCA was carried out on two datasets: one trial set considering only the specimens coming from experimental animals (*n* = 26) and the other where field samples were added as test set (*n* = 93).

## 3. Results

### 3.1. Animal Trial and Collection of Field Samples

Analytical findings from samples collected in the preclinical stage allowed, together with data on the health and welfare of the animals, the correct procedure of the planned clinical trial from day 94 to day 114 (data not shown). During the preclinical trial, one of the animals belonging to the nandrolone-treated group needed to be euthanized due to failure to thrive (day 28, neutered male). The other animals reached an average body weight of 120.9 ± 10.9 kg on the day of slaughter with no significant differences between control (b.w. 122.8 ± 8.5 kg) and exposed groups (b.w. 119.8 ± 8.9 kg for testosterone and b.w. 123.1 ± 13.3 kg for nandrolone groups). The FCR (feed conversion ratio) was calculated for animals weighing between 14 and 90 kg as the ratio of kg of feed consumed per kg of weight gain. For the control group, the average FCR was 2.32; for the nandrolone group, the average FCR was 2.38; and the average FCR was 2.15 for the testosterone group. Comparisons of FCR values between groups of animals were also not statistically significant. Collected specimens are detailed in Table 2.

Regarding the animals selected from the field survey, a total number of 67 liver specimens were collected from Italian slaughterhouses. Relevant data on breed, sex, age, and weight are reported in Supplementary Material S1.

### 3.2. Quality Check of RNA Samples

Extracted total RNA was successfully recovered from the 26 liver specimens collected at the end of the animal trial and from 57 out of the 67 liver samples from field pigs. RNA concentrations ranged between 848 ng/µL and 1968 ng/µL (mean concentration of 1400 ng/µL). RIN values ranged from 7 to 9.5 (mean RIN value 7.5). Further evaluation of RNA integrity was also conducted using DV_200_ calculations (percentage of RNA fragments > 200 base pairs), revealing low fragmentation rates (DV_200_ > 70% in selected extracts) in all selected samples. Detailed data on RNA extracts are reported in Supplementary Material S1.

### 3.3. RNAseq Analysis

**Figure 1:**
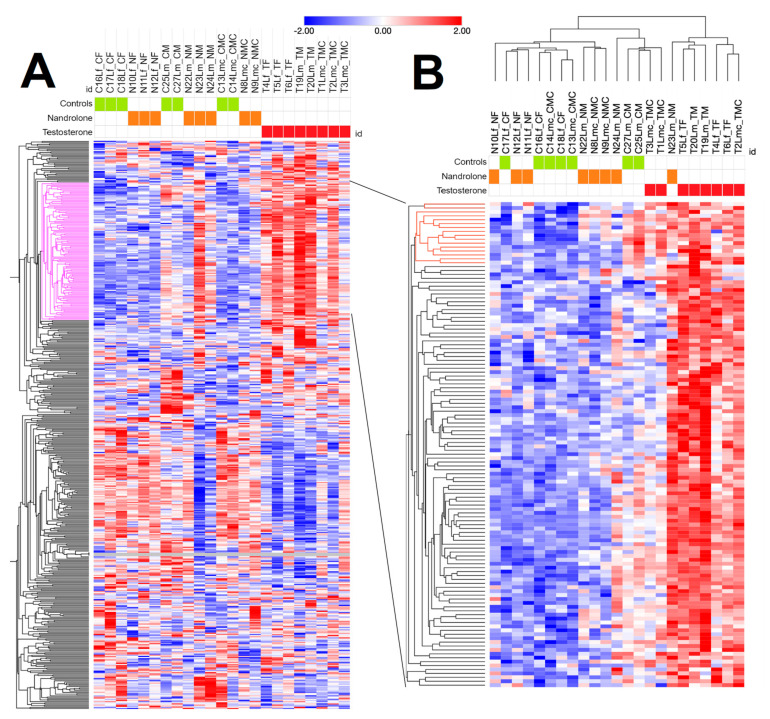
Hierarchical clustering of DEGs. (**A**) Heatmap of the 491 differentially expressed genes depicted from the analysis of Sustanon-treated and Myodine-treated pigs vs control animals. Black frame contains the subset of 143 genes that better discriminate control animals from treated animals. (**B**) Heatmap of the 143 genes on the pink cluster in panel A. Black frame includes the subset of 16 genes (red), providing the best discrimination between treated and control animals.

Comparative transcriptomics analysis, considering both Sustanon-treated and Myodine-treated pigs vs control animals, globally identified 491 differentially expressed genes. These were then used to generate a heatmap (Figure 1).

A discriminant transcriptional signature related to applied treatments was only identified in liver samples from Sustanon-treated pigs, characterizing a cluster of 143 DEGs and, within this, another more restricted cluster of 16 genes that could still discriminate well between untreated gilts, barrows, and boars compared to the relative testosterone ester-treated counterparts.

Genes selected using clustering approaches were: Potassium voltage-gated channel subfamily B member 2 (KCNB2), Inositol 1,4,5-trisphosphate receptor type 3 (ITPR3), Dihydrodiol dehydrogenase (DHDH), Periplakin (PPL), Dehydrogenase/reductase (SDR family) member 4 (DHRS4), Telomeric repeat binding factor 2 (TERF2), Thioesterase superfamily member 5 (THEM5), Mitogen-activated protein kinase 4 (MAPK4), Ankyrin repeat and SOCS box containing 4 (ASB4), Calcium voltage-gated channel subunit alpha1 H (CACNA1H), UDP-GlcNAc:betaGal beta-1,3-N-acetylglucosaminyltransferase 8 (B3GNT8), ATPase Na^+^/K^+^ transporting subunit alpha 2 (ATP1A2), Tubulointerstitial nephritis antigen (TINAG), Dual oxidase 1 (DUOX1), Glycine-N-acyltransferase like 2 (GLYATL2), and Phospholipase B1 (PLB1).

Following heatmap clustering, an enrichment analysis was conducted on the DEG list related to studied treatments in order to verify that, among the most significant gene ontology terms found (BP, MF, and CC), steroid hormone metabolism and related pathways were also enriched.

**Figure 2:**
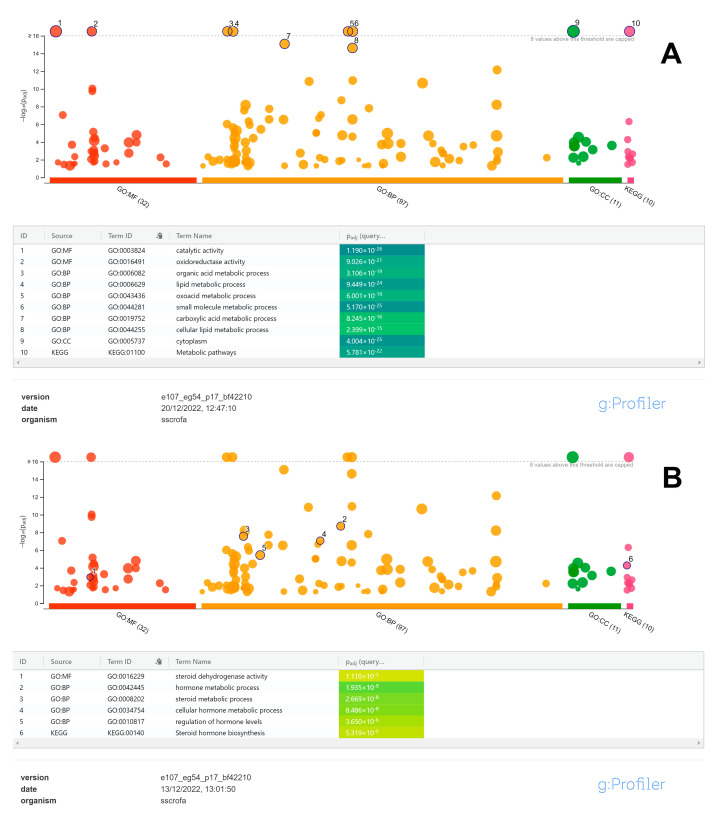
Gene ontology terms related to the most significant molecular functions, biological processes, and cellular components induced by steroid esters in pig livers (**A**). Steroids and hormonal metabolic processes are also induced by treatment with esters (**B**).

The GO enrichment analysis resulted in 137 annotated terms for genes with significant differences between the two groups, including 31 MF, 95 BP, and 11 CC terms, plus ten enriched KEGG pathways (Kyoto Encyclopedia of Genes and Genomes). The most significant top ten GO/KEGG items are reported in Figure 2. These terms are mainly related to metabolic processes and pathways, fat metabolism, and associated catalytic activities located in the cytoplasm. KEGG pathways and other more specifically steroid-related GO terms were also enriched as expected, but with less significant p values when compared to the main metabolic process listed in Figure 2.

**Figure 3:**
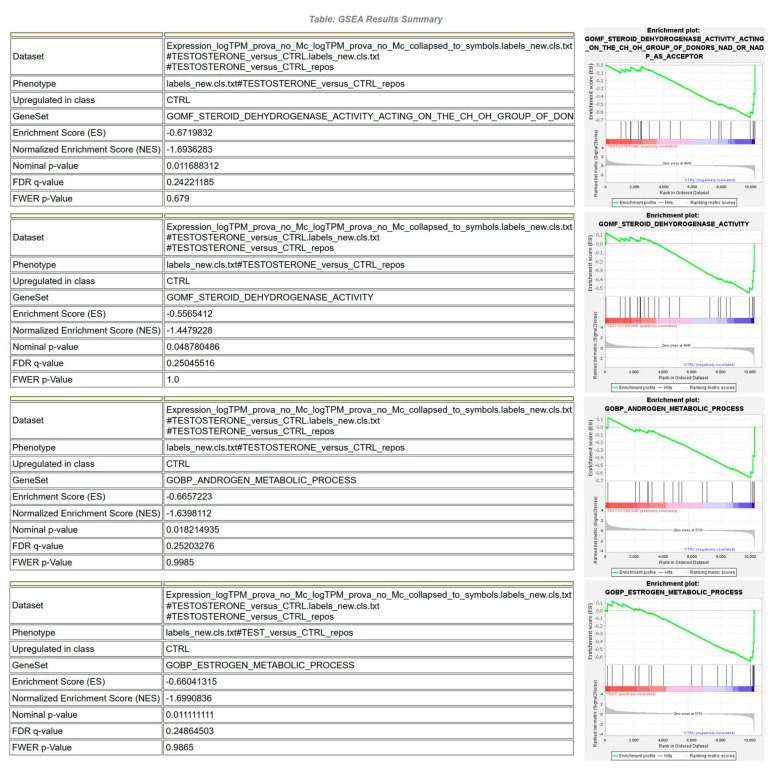
GSEA analysis of steroid ester-related DEGs. Gene identifiers were at first converted into human orthologs using gProfiler orthology search tool (g:Orth).

Gene Set Enrichment Analysis (GSEA), although requiring conversion from pig to human genes using orthologous annotation, was considered in the present study, being an empirical phenotype-based permutation test procedure able to preserve the correlation structures of the gene expression datasets [21]. When compared to gProfiler GO analysis (Figure 2B), GSEA also confirmed, on a reduced number of GO terms, the significant enrichment of specific steroid-related MF and BP pathways (Figure 3).

### 3.4. Real-Time PCR Validation Study

Real-Time PCR validation was performed to confirm significant fold changes in 16 genes shown in Figure 1B. Gene identifiers and primers sequence of both tested reference genes and transcriptional biomarkers are reported in Supplementary Material S2.

**Figure 4:**
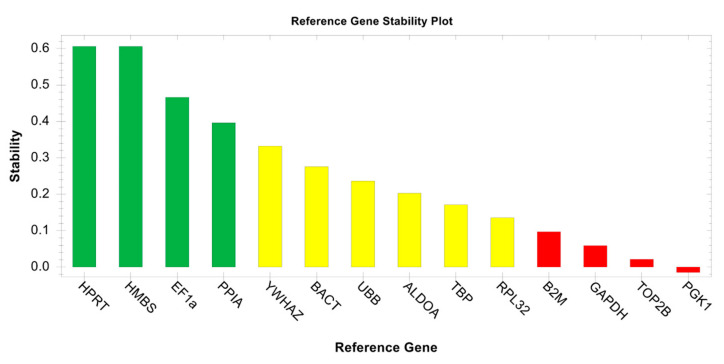
GeNorm evaluation of selected reference genes. HPRT, HMBS, EF1α, and PPIA showed acceptable stability and were therefore selected for normalization of gene expression data using the ΔΔCq method.

At first, the evaluation of primers via dual in silico and in vitro approaches allowed the best primer pairs for each target to be defined (14 reference genes and 16 DEGs). GeNorm evaluation of 14 reference genes then found HPRT, HMBS, EF1α, and PPIA to be the most stable genes across both experimental and field samples (Figure 4).

**Figure 5:**
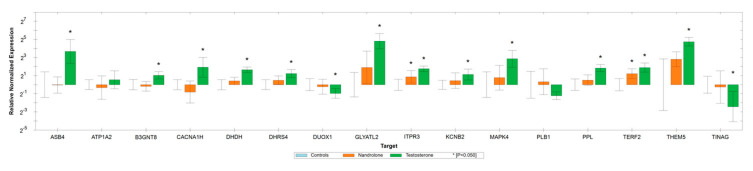
Detailed Fold Changes (±CI at 95%) in selected 16 DEGs identified using RNAseq.

Real-Time PCR allowed the confirmation of the expected fold changes (±confidence intervals at 95%) from RNAseq-identified DEGs via ΔΔCq relative quantification (Figure 5).

**Figure 6:**
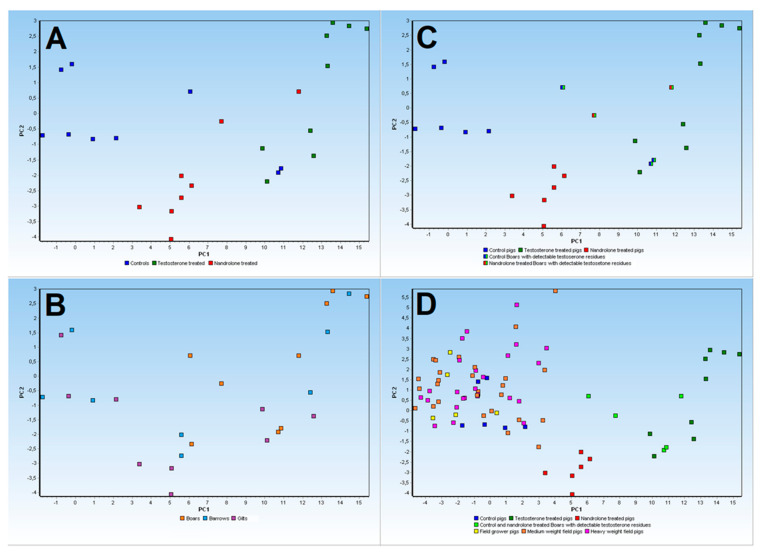
Principal Component Analysis of reference samples from the animal trial (**A**–**C**) and from field samples collected in Italy (**D**).

The PCA analysis based on the collected gene expression profiles in experimental animals is reported in Figure 6A. To gain further insights from recorded PCA results, relevant metadata like sex status (boars, barrows, gilts) were applied to samples clustered in score plots (Figure 6B). Therefore, considering the physiological levels of testosterone found on uncastrated boars belonging to the control group and the nandrolone-treated group (see residue analysis results in Table 3), a rearranged representation of treated and control groups using PCA is reported in Figure 6C.

Finally, a conclusive PCA analysis was performed also considering field samples (where only castrated barrows and gilts were available during abattoir samplings), characterized by gene expression profiles on the same 16 biomarkers considered on experimental pigs’ liver samples (Figure 6D). Scores and loadings of performed PCA are reported in Supplementary Material S3.

### 3.5. Analysis of Steroid Residues (Experimental Animals)

Steroid profiles in blood serum samples collected from experimental animals are reported in Table 3. Mean testosterone levels recorded in the treated group were 19.97 ± 8.58 ng/mL (*n* = 9). Mean nandrolone levels recorded in the treated group were 7.79 ± 2.66 ng/mL (*n* = 8). Traces of estradiol were found in a few samples of animals from both treated and control groups. Low concentrations of testosterone and nandrolone were determined in boars from the control group (see Table 3).

### 3.6. Analysis of Steroid Residues (Field Animals)

The method used, originally developed in bovine serum [29], met the criteria set out in Commission Decision 2002/657/EC for the purpose of both screening and confirmation, resulting in being specific, sensitive, and suitable for measuring the natural level of sex hormones in pig blood serum.

In all 57 serum samples, 17β-testosterone was not detected above the Limit of Quantification (LOQ) value.

In twenty serum samples, progesterone was detected at concentration levels above the LOQ value, five of which were barrows and fifteen of which were gilts. Recorded levels of progesterone were in line with data already reported in other recent abattoir surveys [30].

In only one female serum sample, 17β-estradiol was detected near the LOQ level. 

The detailed results of the quantitative method are described in Table 4.

## 4. Discussion

In the present work, an investigation of the coding transcriptome of liver samples collected from experimental and field pigs was performed to identify transcriptional signatures specifically related to the administration of testosterone and nandrolone esters. These illicit practices are known to be difficult to counter because endogenous steroid residues may sometimes be present in different animal matrices/specimens [10,31]; these aspects, together with the known relations among different “natural” steroids and metabolites, have always hampered the development of a definitive approach for control [32]. Therefore, the indirect detection of steroid ester abuse using untargeted biological methods still represents a chance to focus further investigations and, when needed, confirmatory analysis [15,33].

To achieve the aim of the study, transcriptional markers were identified using conventional treated vs control comparisons, in which uncastrated boars were included in both control and treated groups, together with castrated male pigs (barrows) and female pigs (gilts). This choice, apparently not very useful or even counterproductive, was originally made to select biomarkers unresponsive to a potential source of confounding variance caused by the “natural” occurrence of endogenous testosterone and/or nandrolone background levels in pigs, trying therefore to identify any transcriptional perturbation strictly related to synthetic steroid ester administration only (in our case Sustanon and Myodine). Finally, the field survey performed in Italy, including different weight categories, different breeds, and different types of pig farming, was designed to attempt a reliable assessment of the most critical sources of inter-individual and biological variability, which are known to affect the sensitivity and the specificity of indirect biological methods [32]. For this aspect, the concomitant steroid profiles of testosterone, estradiol, and progesterone, determined in pig serum samples according to a fit-for-purpose validated method compliant to Commission Regulation (EU) 2021/808, allowed further verification in a real field context of the specificity of selected biomarkers. The developed multi-residue method will need to be extended to other steroids like nandrolone, which is known to occur in both domestic and wild boars [10,34,35].

RNAseq analysis identified up to 491 DEGs related, as expected, to biological processes, molecular functions, and KEGG pathways (Figure 2 and Figure 3) that are known to be influenced by sex steroids in other farm species [36,37]. Among the DEGs found using the preliminary heatmap clustering analysis of RNAseq data, a minimum panel of 16 transcripts were still able to differentiate control animals from treated ones (Figure 1). Confirmation analysis was conducted using Real-Time PCR (Figure 6), a lower-throughput technique compared to RNAseq, but characterized by higher dynamic range and quantification accuracy. Collected quantification data (Figure 5) successfully confirmed significant differential expression in fourteen out of sixteen genes (*p* < 0.05), comparing testosterone-treated (Sustanon) and control pig groups, while only two out of sixteen genes were differentially expressed, according to univariate analysis, when comparing nandrolone-treated (Myodine) and control samples (Figure 5). Moreover, the transcriptional signature related to Myodine treatment, identified using RNAseq, was characterized by a larger number of downregulated genes in comparison to the Sustanon signature (see B3GNT8, ATP1A2, CACNA1H, DUOX1, and TINAG profiles in Figure 5). As already seen in previous studies based on the profiling of transcriptional biomarkers to unveil growth promoters’ administration in farm animals [38], the validation analysis of RNAseq/Microarray data using Real Time and/or Digital PCR has often revealed how dowregulated genes, especially those that are characterized by low basal expression levels, are more difficult to validate for reliable application in field/unknown samples, where optimal preanalytical conditions in sampling procedures are not always guaranteed [39].

Indeed, subsequent PCA models (Figure 6A–D) showed that Myodine-treated pigs (red dots) were only slightly different from control pigs (blue dots) compared with Sustanon-treated animals (dark-green dots). Moreover, multivariate analysis also revealed that uncastrated boars belonging to both control and nandrolone-treated groups were clustered nearer to or together with testosterone-treated barrows, gilts, and boars (see the five red/light-green and blue/light-green dots in Figure 6C). These findings were confirmed by the steroid residue profiles collected on respective serum samples (five boars with a mean testosterone concentration of 1.68 ng/mL; see Table 3), showing that low endogenous levels of testosterone could be present in uncastrated boars, which could consequently affect the liver gene expression profiles. 

In light of the recorded results, considering that intact (uncastrated) boars are not usually intended for human consumption and that the rates of partially intact pigs (e.g., monorchid, incorrectly neutered barrows, etc.) are relatively low, the potential application of selected transcriptional biomarkers to setup bio-based screening methods was then evaluated in pig liver samples collected through field survey.

The gene expression profiles from animals sampled in Italian slaughterhouses according to different age and weight categories (see Supplementary Table S1) were studied using PCA analysis, and they tended to all be clustered around the samples of control pigs from the experimental trial (Figure 6D). These results were further confirmed by residue profiles in respective serum samples, in which no field animals were found positive for testosterone residues (Table 4).

As already shown in Figure 6C, the five experimental animals with reported low testosterone levels (mean concentration of 1.68 ng/mL) were grouped using PCA, mainly nearer to or together with testosterone-treated pigs (light-green dots, Figure 6D).

The tested panel of transcriptional biomarkers has therefore confirmed, at least for the illicit administration of testosterone esters, an interesting potential for supplementary field investigations, which will need to be extended in order to be validated on an even larger number of pig specimens than that already shown in the present study. In this regard, more refined supervised classification tools [38,39,40] will need to be considered in light of ISO-oriented validation approaches based on multivariate analysis [41].

## 5. Conclusions

The disclosure of illicit doping practices in farmed animals is still difficult in European countries due to the misuse of several classes of both “old and forgotten” or “new and unknown” drugs combined with more and more refined administration schedules on animals. Considering the recent choices of EU countries to set up risk-based monitoring plans, designed to potentially have the flexibility needed to effectively contrast these practices [42], the untargeted methods will represent an ideal tool to contrast animal doping, being based on omic approaches that could allow both retrospective analysis and the refinement of existing targeted strategies [43]. In this monitoring framework, the use of identified transcriptional biomarkers has confirmed how the transcriptomics approach can be a useful tool for the indirect detection of the misuse of anabolic steroids in fattening pigs.

## Figures and Tables

**Table 1:** Mass spectrometry parameters applied. (Abbreviations: DP—Declustering Potential; EP—Entrance Potential; CE—Collision Energy; CXP—Collision Exit Potential).

Analytes	DP	EP	Parent Ion	Product Ion	CE	CXP
17β-estradiol-d4	−130	−10	275.2	147.0	−53	−12
				187.0	−54	
				145.0	−65	
17β-estradiol	−100	−10	271.2	145.0	−50	−12
				183.1	−54	
				143.0	−67	
17β-testosterone-d3	90	10	292.2	256.4	25	12
				109	32	
				97.2	29	
17β-testosterone	74	10	289.1	97.2	29	12
				109.0	33	
				79.1	70	
Progesterone-d9	80	10	324.3	100.1	29	12
				113.1	33	
				306.4	25	
Progesterone	103	10	315.2	97.1	26	12
				109.1	30	
				297.4	22	

**Table 2:** Animal trial group compositions, identifiers, and respective sample labelling (barrow: castrated/neutered pig, gilt: female swine that has not yet produced a litter, boar: uncastrated male pig).

Group	Pig No.	Sex	Liver	Serum
Testosterone	1	barrow	T1L-Mc	S1
	2	barrow	T2L-Mc	S2
	3	barrow	T3L-Mc	S3
	4	gilt	T4L-F	S4
	5	gilt	T5L-F	S5
	6	gilt	T6L-F	S6
	19	boar	T19L-M	S19
	20	boar	T20L-M	S20
	21	boar	T21L-M	S21
Nandrolone	8	barrow	N8L-Mc	S8
	9	barrow	N9L-Mc	S9
	10	gilt	N10L-F	S10
	11	gilt	N11L-F	S11
	12	gilt	N12L-F	S12
	22	boar	N22L-M	S22
	23	boar	N23L-M	S23
	24	boar	N24L-M	S24
Control	13	barrow	C13L-Mc	S13
	14	barrow	C14L-Mc	S14
	15	barrow	C15L-Mc	S15
	16	gilt	C16L-F	S16
	17	gilt	C17L-F	S17
	18	gilt	C18L-F	S18
	25	boar	C25L-M	S25
	26	boar	C26L-M	S26
	27	boar	C27L-M	S27

**Table 3:** Profiles of steroid residues in serum samples.

Group	Sample ID	Sex	Testosterone	Nandrolone	Estradiol
			Concentration (ng/mL)	Concentration (ng/mL)	Concentration (ng/mL)
Testosterone	S1	Barrow	15.85	0	0
	S2	Barrow	13.71	0	0
	S3	Barrow	17.48	0	0
	S4	Gilt	16.79	0	0
	S5	Gilt	21.20	0	0
	S6	Gilt	41.99	0	0.014
	S19	Boar	15.99	0	0
	S20	Boar	20.53	0	0
	S21	Boar	16.17	0	0
Nandrolone	S8	Barrow	0	7.32	0
	S9	Barrow	0	4.67	0
	S10	Gilt	0	5.71	0
	S11	Gilt	0	8.49	0
	S12	Gilt	0	5.47	0
	S22	Boar	0	10.72	0
	S23	Boar	1.19	7.59	0
	S24	Boar	1.17	12.35	0
Control	S13	Barrow	0	0	0
	S14	Barrow	0	0	0
	S15	Barrow	0	0	0
	S16	Gilt	0	0	0
	S17	Gilt	0	0	0.044
	S18	Gilt	0	0	0
	S25	Boar	1.97	0	0.022
	S26	Boar	1.91	0.90	0.031
	S27	Boar	2.15	3.93	0

**Table 4:** Analysis of field survey residues in collected blood serum samples. Testosterone levels in all tested samples were <LOQ.

Animal ID	Sex	Breed	Weight Range for Batch (Kg)	Weight Category	Age (Months)	Progesterone (ng/mL)	Estradiol (ng/mL)
1	Barrow	Black Pig of Piedmont	90	medium	8	1.40	0
2	Gilt	Black Pig of Piedmont	90	medium	8	15.22	0
3	Gilt	Large White	150–160	heavy	10	21.76	0
4	Gilt	Large White	150–160	heavy	10	31.24	0
6	Gilt	Large White	150–160	heavy	10	2.40	0
7	Barrow	Large White	150–160	heavy	10	0	0
8	Gilt	Unknown commercial crossbred	100–110	medium	10	0.62	0
9	Gilt	Unknown commercial crossbred	100–110	medium	10	0	0
10	Gilt	Unknown commercial crossbred	100–110	medium	10	28.08	0
11	Gilt	Unknown commercial crossbred	100–110	medium	10	15.38	0
12	Barrow	Large White × Landrace	150–160	heavy	11	0	0
15	Barrow	Large White × Landrace	150–160	heavy	11	0	0
16	Barrow	Large White × Landrace	150–160	heavy	11	0	0
17	Barrow	Large White × Landrace	150–160	heavy	11	0	0
18	Barrow	Large White × Landrace	150–160	heavy	11	0	0
19	Barrow	Large White × Landrace	150–160	heavy	11	0	0
20	Barrow	Large White × Landrace	150–160	heavy	11	0	0
21	Barrow	Large White × Landrace	150–160	heavy	11	0	0
23	Gilt	Large White	110–120	medium	10	0	0
24	Gilt	Large White	110–120	medium	10	0	0
25	Gilt	Large White	110–120	medium	10	0.51	0
26	Barrow	Large White	110–120	medium	10	1.21	0
27	Gilt	Black Pig of Piedmont	90–100	medium	8	0	0
28	Barrow	Black Pig of Piedmont	90–100	medium	8	0.75	0
29	Barrow	Black Pig of Piedmont	90–100	medium	8	0.45	0
30	Gilt	Black Pig of Piedmont	90–100	medium	8	0	0
31	Barrow	Black Pig of Piedmont × Large White	100–110	medium	8	0	0
32	Barrow	Black Pig of Piedmont × Large White	100–110	medium	8	0	0
33	Gilt	Black Pig of Piedmont × Large White	100–110	medium	8	18.94	0
35	Barrow	Landrace	170–180	heavy	9	0	0
36	Barrow	Landrace	170–180	heavy	9	0	0
37	Barrow	Landrace	170–180	heavy	9	0	0
38	Barrow	Landrace	170–180	heavy	9	0	0
39	Barrow	Landrace	170–180	heavy	9	0	0
41	Barrow	Landrace	170–180	heavy	9	0	0
43	Barrow	Landrace	170–180	heavy	9	0	0
44	Gilt	Unknown commercial crossbred	150–160	heavy	10	8.04	0
46	Gilt	Unknown commercial crossbred	150–160	heavy	10	0	0.02
47	Gilt	Unknown commercial crossbred	150-–160	heavy	10	26.08	0
50	Barrow	Unknown commercial crossbred	150–160	heavy	10	0	0
51	Barrow	Unknown commercial crossbred	150–160	heavy	10	0	0
52	Gilt	Unknown commercial crossbred	150–160	heavy	10	5.72	0
53	Gilt	Duroc	60–70	small	6	4.41	0
54	Gilt	Duroc	60–70	small	6	0	0
55	Barrow	Duroc	60–70	small	6	0	0
56	Gilt	Duroc	60–70	small	6	0.66	0
57	Gilt	Duroc	60–70	small	6	0	0
58	Barrow	Black Pig of Sicily	70–90	medium	8	0	0
59	Barrow	Black Pig of Sicily	70–90	medium	8	0	0
60	Gilt	Black Pig of Sicily	70–90	medium	8	0	0
61	Barrow	Black Pig of Sicily	70–90	medium	8	0	0
62	Gilt	Black Pig of Sicily	70–90	medium	8	0	0
63	Barrow	Black Pig of Sicily	70–90	medium	8	0	0
64	Gilt	Black Pig of Sicily	70-90	medium	8	18.97	0
65	Barrow	Black Pig of Sicily	70–90	medium	8	0	0
66	Barrow	Black Pig of Sicily	70–90	medium	8	0	0
67	Barrow	Black Pig of Sicily	70–90	medium	8	0.48	0

## Data Availability

RNAseq data are available in the Gene Expression Omnibus (GEO) data repository under the accession code GSE233177. Other data are available on request from the authors.

## Supplementary Materials

The following supporting information can be downloaded at: https://www.mdpi.com/article/10.3390/ani13223495/s1, Table S1: Relevant data on breed, sex, age, and weight of pigs selected from Italian slaughterhouses; Table S2: Gene identifiers and primers sequence of reference genes and transcriptional biomarkers validated through qPCR; Table S3: Scores and loadings of performed PCA.
