# Supplementary material for: RNAseq Analysis of Livers from Pigs Treated with Testosterone and Nandrolone Esters: Selection and Field Validation of Transcriptional Biomarkers
Source: Animals (Basel). 2023 Nov 13;13(22):3495. doi: 10.3390/ani13223495 (PMC10668810; doi:10.3390/ani13223495)
Supplement: Supplementary file 1 [file animals-13-03495-s001.zip › Supplementary Material S2.pdf]

Supplementary Material S2: primer sequences, identifiers and ensemble ID of reference genes and DEG.

Gene name	Ensembl ID	Primer Sequences (5'-3')	
		Forward	Reverse
EF1A	ENSSSCG00000004489	5'- CAC ACT GCT TGA AGC TCT GGA T -3'	5'- GAC TGT ACC AAT ACC ACC AAT TTT GT -3'
B2M	ENSSSCG00000051899	5'- TCC ACA CTG AGT TCA CTC CTA ACG -3'	5'- TGG TCT CGA TCC CAC TTA ACT ATC T -3'
HPRT1	ENSSSCG00000034896	5'- ATC ATT ATG CCG AGG ATT TGG A -3'	5'- CCT CCC ATC TCT TTC ATC ACA TC -3'
HMBS	ENSSSCG00000015108	5'- GAG GCT GCA GTG TGC CAG TAG -3'	5'- TGC ATG CTC TCT GCA CCA TT -3'
YWHAZ	ENSSSCG00000006062	5'- GCT GGT GAT GAT AAG AAA GGG ATT -3'	5'- TGA TAG GAT GTG TTG GTT GCA TTT -3'
β-ACT	ENSSSCG00000007585	5'- CCA ACT GGG ACG ACA TGG A -3'	5'- CCA ACT GGG ACG ACA TGG A -3'
UBB	ENSSSCG00000018033	5'- TGC ATT TTG ACC TGT GAG TGA AG -3'	5'- GAG GGT GAT TCG GGT GTG AT -3'
ALDOA	ENSSSCG00000032556	5'- CCA CGA GAC GCT CTA CCA GAA -3'	5'- CAC ACC CTT GTC CAC CTT GAT -3'
TBP	ENSSSCG00000037372	5'- CTA TAA GGT TAG AAG GCC TTG TGC TT -3'	5'- CAA TTC TCG GTT TGA TCA TTC TGT -3'
RLP32	ENSSSCG00000035811	5'- TCT GGT ACA CAA TGT AAA AGA GCT TGA -3'	5'- GGC TTT GCG GTT CTT GGA A -3'
GAPDH	ENSSSCG00000000694	5'- CCA TCT TCC AGG AGC GAG ATC -3'	5'- GCC TTC TCC ATG GTC GTG AA -3'
TOP2B	ENSSSCG00000011213	5'- CAA CAA GCA AGA AAC CGA AGA A -3'	5'- GGT CCG TGG CAG AGA AGG T -3'
PGK1	ENSSSCG00000012440	5'- CCA CTG TGG CCT CTG GTA TAC C -3'	5'- AAT CTG CTT AGC CCG AGC AA -3'
PPIA	ENSSSCG00000016737	5'- CAT ACG GGT CCT GGC ATC TT -3'	5'- TGC CAT CCA ACC ACT CAG TCT -3'
KCNB2	ENSSSCG00000006185	5'-GGT GTG CGA CGA CTA CAA TCT G-3	5'-TCC CGG ATG GCG ATC A-3'
ITPR3	ENSSSCG00000001518	5'-CAC GAG AAC CAG ACC TGC AT-3'	5'-TCG TTG AGA ATC AGC GCA GT-3'
DHDH	ENSSSCG00000003144	5'-AAA CAC GAC ATC CCC AAG GC-3	5'-GCC AAT ATA GGC CAC CTC CAC-3'
PPL	ENSSSCG00000007927	5'-GGC CGC ATG CAG TAT GAC T-3'	5'-TCC GGT TGA TGA AAT TCT CGT A-3'
DHRS4	ENSSSCG00000002013	5'-GGC CAC AGT CCT GAT GAC AA-3'	5'-AGC CGC CTC CTC GTT TCT-3'
TERF2	ENSSSCG00000002762	5'-GGA TGA TGC AGA GCC CTA CCT-3'	5'-AGT GGA GGA CTC AGA TTT CAA AGC-3'
THEM5	ENSSSCG00000006614	5'-CCA TCC TGG TGC CCT CAA C-3'	: 5'-CCA TCC TGG TGC CCT CAA C-3'
MAPK4	ENSSSCG00000004520	5'-ACC TTG GTG GGC GTT TCA-3'	5'-AGC CCA TTG ACG CCA AAG-3'
ASB4	ENSSSCG00000015333	5'-TCT GTA CAA CTC CAA GCT CCA TTC-3'	5'-CAT TCG CCC CTC TCC AAA-3'
CACNA1H	ENSSSCG00000034266	5'-TCA TCA TCG TGG GTT CCT TTT T-3'	5'-AGA ACT GCG TGG CGA TCA C-3'
B3GNT8	ENSSSCG00000032536	5'-CCC CAA CTG TGC TCC ACA A-3'	5'-CTG GAA CAG GTA AAG TCG GAT GT-3'
ATP1A2	ENSSSCG00000006391	5'-TGG GCA GGA GAA CAT CTC TGT-3'	5'-AGC TGA CTC GGA GGC ATC A-3'
TINAG	ENSSSCG00000001484	5'-AAA ACT GCA ATT CCT GCA CAT G-3'	5'-GGC ATA CGT GCT GGG AAC A-3'
DUOX1	ENSSSCG00000004675	5'-TTC TCC CAG GTG CTA GAC ATT GA-3'	5'-CCC GCA CCT TCT GTG ATG A-3'
GLYATL2	ENSSSCG00000013148	5'-GGC ATG CCC CCA GGT TA-3'	5'-CTC TTG GCA ACC TTG GAT CTG-3'
PLB1	ENSSSCG00000034756	5'-GCA GGC CAG TGT TTT CTG TAA CT-3'	5'-TCC AAC CTG GCC AGC TCT T-3'
