# Supplementary material for: RNAseq Analysis of Livers from Pigs Treated with Testosterone and Nandrolone Esters: Selection and Field Validation of Transcriptional Biomarkers
Source: Animals (Basel). 2023 Nov 13;13(22):3495. doi: 10.3390/ani13223495 (PMC10668810; doi:10.3390/ani13223495)
Supplement: Supplementary file 1 [file animals-13-03495-s001.zip › Supplementary Material S3.pdf]

Biomarkers	Loadings1	Loadings2	Loadings3	Loadings4
**PLB1**	-0.183873472	0.091546563	-0.453692066	0.38098242
**TINAG**	-0.229264152	-0.514081185	0.54343168	0.170719222
**GLYATL2**	0.451095634	0.017354242	-0.245970326	0.326975271
**DHDH**	0.126581383	0.040098582	0.194652864	0.063091614
**DHRS4**	0.13181142	0.013676545	0.066821181	-0.045263081
**DUOX1**	-0.105185263	0.021544789	-0.092336239	0.155794671
**ITPR3**	0.155711761	-0.060804913	0.136664462	0.196253741
**KCNB2**	0.093267699	0.06659897	0.24616929	-0.041247083
**B3GNT8**	0.08912428	0.131773401	0.001670945	0.002787618
**PPL**	0.168174812	-0.005323987	0.142392076	0.157691785
**TERF2**	0.197795239	-0.083176668	-0.011883304	-0.026684212
**MAPK4**	0.297499872	0.107751599	-0.167013054	0.120321899
**THEM5**	0.583250971	-0.5540733	-0.115956695	-0.199723146
**ASB4**	0.327256014	0.379599903	0.323877074	-0.106481494
**ATP1A2**	0.047571748	-0.074676894	0.186550255	0.744092503
**CACNA1H**	0.139064266	0.473345037	0.326696577	0.057543129

Animal Trial Samples	Scores1	Scores2
**C13L_Mc**	-0.2053113	1.592660338
**T1L_Mc**	13.3221576	1.533550971
**C26L_M**	6.053190535	0.703495055
**N24L_M**	7.715030173	-0.253567634
**C27L_M**	10.71036833	-1.912537328
**T21L_M**	13.57698224	2.94032204
**N8_Mc**	5.599161873	-2.015165618
**C14L_Mc**	-1.744044948	-0.7106748
**N10L_F**	5.061944624	-3.166738739
**C16L_F**	-0.358256574	-0.67796488
**N11L_F**	5.038578058	-4.069145451
**C18L_F**	-0.749416591	1.416567922
**N22L_M**	6.131807385	-2.341891347
**T19L_M**	13.27768715	2.512547423
**C15L_Mc**	0.909521393	-0.824608143
**T2L_Mc**	12.40978388	-0.554231296
**T3L_Mc**	9.87052509	-1.130373458
**N9L_Mc**	5.591708635	-2.730507283
**T4L_F**	10.11364459	-2.206708577
**C17L_F**	2.147508019	-0.795980438
**T5L_F**	14.43620521	2.838344164
**C25L_M**	10.86119652	-1.784356545
**N12L_F**	3.371181254	-3.0321378
**T6L_F**	12.56826351	-1.373752482
**T20L_M**	15.38193903	2.742270802
**N23L_M**	11.79314348	0.707563332

Field Animal Samples	Scores1	Scores2
**1**	4.002869372	5.819442318
**2**	0.926320385	1.557744495
**3**	1.082888329	2.674268593
**4**	1.602084284	3.208882463
**6**	1.634990392	5.144615755
**7**	1.767731055	0.457814772
**8**	3.46802185	3.035573388
**9**	0.666211178	0.780532906
**10**	1.575287827	4.07533292
**11**	-3.122195542	1.852358546
**12**	-0.753101595	0.925675017

**15**	-0.8962193	1.953399535
**16**	-2.069484058	0.906353118
**17**	-0.43152303	1.639778717
**18**	2.992406484	2.316012778
**19**	-0.755605868	0.802926644
**20**	-1.750005665	3.504675401
**21**	2.070235054	-0.609049415
**23**	-1.581313736	0.607525765
**24**	-4.307729802	0.630001696
**25**	1.199919129	0.619569401
**26**	1.095528076	-1.071816652
**27**	3.240199971	-0.463552169
**28**	-4.37667212	1.078004832
**29**	0.756739618	1.22651489
**30**	-3.518505362	0.207243131
**31**	-4.644803438	0.113903223
**32**	-3.509028185	2.48878355
**33**	-3.193900324	0.427810963
**35**	-1.953109172	2.604448828
**36**	-4.447065138	1.539901383
**37**	-0.419182649	-0.246355716
**38**	-0.794569507	0.709161594
**39**	-0.932564025	1.063822124
**41**	-1.6803511	0.58407209
**43**	-3.75426337	0.956982694
**44**	-3.428692633	-0.750961027
**46**	-2.273193463	-0.584586211
**47**	-3.879341144	0.509632326
**50**	-1.910647327	2.450113604
**51**	-1.456883285	3.861154581
**52**	-2.075618451	0.169797476
**53**	-2.146362018	-0.203881432
**54**	-2.656381108	1.744083327
**55**	-3.558901474	-0.363859674
**56**	-2.473833371	2.825071997
**57**	0.401835752	-0.097875175
**58**	-1.097389247	1.709630005
**59**	2.984766852	-1.756568644
**60**	0.086662677	-0.029615183
**61**	-0.96267981	2.101203236
**62**	3.342344697	1.964644557
**63**	-3.196646377	1.475699414
**64**	-0.804045674	0.747860482
**65**	-3.370722208	2.457882436
**66**	-3.249818947	1.297978975
**67**	1.759075579	-0.447751666
