# Supplementary material for: RNAseq Analysis of Livers from Pigs Treated with Testosterone and Nandrolone Esters: Selection and Field Validation of Transcriptional Biomarkers
Source: Animals (Basel). 2023 Nov 13;13(22):3495. doi: 10.3390/ani13223495 (PMC10668810; doi:10.3390/ani13223495)
Supplement: Supplementary file 1 [file animals-13-03495-s001.zip › Supplementary_Material_S1.pdf]

Year	Sampling ID	Sex	Breed	Weight range for batch (Kg)	Weight category	Age (months)	Slaughter plant location	[RNA] ng/µL	RIN	DV200
2021	1	Barrow	Black Pig of Piedmont	90	medium	8	Piedmont	1180	7.3	85%
2021	2	Gilt	Black Pig of Piedmont	90	medium	8	Piedmont	1000	8.2	82%
2021	3	Gilt	Large white	150-160	heavy	10	Piedmont	896	7.9	84%
2021	4	Gilt	Large white	150-160	heavy	10	Piedmont	1296	9.3	85%
2021	5	Barrow	Large white	150-160	heavy	10	Piedmont	1492	2.9	51%
2021	6	Gilt	Large white	150-160	heavy	10	Piedmont	1196	7.3	71%
2021	7	Barrow	Large white	150-160	heavy	10	Piedmont	1564	7	84%
2021	8	Gilt	Unknown commercial crossbred	100-110	medium	10	Piedmont	852	7.4	75%
2021	9	Gilt	Unknown commercial crossbred	100-110	medium	10	Piedmont	1316	7	78%
2021	10	Gilt	Unknown commercial crossbred	100-110	medium	10	Piedmont	568	9.3	86%
2021	11	Gilt	Unknown commercial crossbred	100-110	medium	10	Piedmont	1,768	7.4	75%
2021	12	Barrow	Large white x Landrance	150-160	heavy	11	Piedmont	1448	7.6	88%
2021	13	Barrow	Large white x Landrance	150-160	heavy	11	Piedmont	<50	N.A.	N.A.
2021	14	Barrow	Large white x Landrance	150-160	heavy	11	Piedmont	125	4.6	55%
2021	15	Barrow	Large white x Landrance	150-160	heavy	11	Piedmont	1100	7	77%
2021	16	Barrow	Large white x Landrance	150-160	heavy	11	Piedmont	1,416	8.9	84%
2021	17	Barrow	Large white x Landrance	150-160	heavy	11	Piedmont	1960	7.1	86%
2021	18	Barrow	Large white x Landrance	150-160	heavy	11	Piedmont	704	9.3	89%
2021	19	Barrow	Large white x Landrance	150-160	heavy	11	Piedmont	852	9.3	81%
2021	20	Barrow	Large white x Landrance	150-160	heavy	11	Piedmont	704	9.5	88%
2021	21	Barrow	Large white x Landrance	150-160	heavy	11	Piedmont	688	9.5	89%
2021	22	Barrow	Large white	110-120	medium	10	Piedmont	56	4.5	52%
2021	23	Gilt	Large white	110-120	medium	10	Piedmont	948	8.1	85%
2021	24	Gilt	Large white	110-120	medium	10	Piedmont	296	8.5	89%
2021	25	Gilt	Large white	110-120	medium	10	Piedmont	604	9.6	89%
2021	26	Barrow	Large white	110-120	medium	10	Piedmont	572	9.1	85%
2021	27	Gilt	Black Pig of Piedmont	90-100	medium	8	Piedmont	1948	7.9	83%
2021	28	Barrow	Black Pig of Piedmont	90-100	medium	8	Piedmont	1792	8.4	81%
2021	29	Barrow	Black Pig of Piedmont	90-100	medium	8	Piedmont	2160	7.2	83%
2021	30	Gilt	Black Pig of Piedmont	90-100	medium	8	Piedmont	1200	7.1	85%
2021	31	Barrow	Black pig of Piedmont x Large white	100-110	medium	8	Piedmont	1688	7.4	83%
2021	32	Barrow	Black pig of Piedmont x Large white	100-110	medium	8	Piedmont	1968	8	80%
2021	33	Gilt	Black pig of Piedmont x Large white	100-110	medium	8	Piedmont	2120	7.2	82%
2021	34	Barrow	Landrace	170-180	heavy	9	Puglia	942	4.9	61%
2021	35	Barrow	Landrace	170-180	heavy	9	Puglia	2850	7	74%
2021	36	Barrow	Landrace	170-180	heavy	9	Puglia	2670	7.8	71%

2021	37	Barrow	Landrace	170-180	heavy	9	Puglia	2340	8	81%
2021	38	Barrow	Landrace	170-180	heavy	9	Puglia	2020	7.2	73%
2021	39	Barrow	Landrace	170-180	heavy	9	Puglia	1724	8.1	80%
2021	40	Barrow	Landrace	170-180	heavy	9	Puglia	<50	N.A.	N.A.
2021	41	Barrow	Landrace	170-180	heavy	9	Puglia	1112	7	75%
2021	42	Barrow	Landrace	170-180	heavy	9	Puglia	520	3.3	51%
2021	43	Barrow	Landrace	170-180	heavy	9	Puglia	956	7.1	71%
2022	44	Gilt	Unknown commercial crossbred	150-160	heavy	10	Piedmont	1520	7.3	81%
2022	45	Barrow	Unknown commercial crossbred	150-160	heavy	10	Piedmont	300	3.6	35%
2022	46	Gilt	Unknown commercial crossbred	150-160	heavy	10	Piedmont	1028	7.8	78%
2022	47	Gilt	Unknown commercial crossbred	150-160	heavy	10	Piedmont	1252	7.1	79%
2022	48	Barrow	Unknown commercial crossbred	150-160	heavy	10	Piedmont	205	4	75%
2022	49	Barrow	Unknown commercial crossbred	150-160	heavy	10	Piedmont	92	3.9	54%
2022	50	Barrow	Unknown commercial crossbred	150-160	heavy	10	Piedmont	880	7.7	90%
2022	51	Barrow	Unknown commercial crossbred	150-160	heavy	10	Piedmont	1008	7.9	78%
2022	52	Gilt	Unknown commercial crossbred	150-160	heavy	10	Piedmont	1604	7.5	84%
2023	53	Gilt	Duroc	60-70	small	6	Piedmont	2586	7.5	75%
2023	54	Gilt	Duroc	60-70	small	6	Piedmont	2768	7.1	78%
2023	55	Barrow	Duroc	60-70	small	6	Piedmont	1824	7.1	71%
2023	56	Gilt	Duroc	60-70	small	6	Piedmont	3240	7	80%
2023	57	Gilt	Duroc	60-70	small	6	Piedmont	1048	7.3	78%
2023	58	Barrow	black pig of Sicily	70-90	medium	8	Sicily	1368	7.1	80%
2023	59	Barrow	black pig of Sicily	70-90	medium	8	Sicily	796	7.3	72%
2023	60	Gilt	black pig of Sicily	70-90	medium	8	Sicily	1100	7	90%
2023	61	Barrow	black pig of Sicily	70-90	medium	8	Sicily	1720	7.1	79%
2023	62	Gilt	black pig of Sicily	70-90	medium	8	Sicily	940	7.4	74%
2023	63	Barrow	black pig of Sicily	70-90	medium	8	Sicily	668	7.7	77%
2023	64	Gilt	black pig of Sicily	70-90	medium	8	Sicily	410	7.1	70%
2023	65	Barrow	black pig of Sicily	70-90	medium	8	Sicily	1196	8.2	73%
2023	66	Barrow	black pig of Sicily	70-90	medium	8	Sicily	780	7	77%
2023	67	Barrow	black pig of Sicily	70-90	medium	8	Sicily	454	7.1	76%

Sample with RIN<7 removed from the study
